# MBD2 Regulates Th17 Cell Differentiation and Experimental Severe Asthma by Affecting IRF4 Expression

**DOI:** 10.1155/2017/6249685

**Published:** 2017-07-20

**Authors:** Aijun Jia, Yueling Wang, Wenjin Sun, Bing Xiao, Yan Wei, Lulu Qiu, Lin Mu, Li Xu, Jianmin Li, Xiufeng Zhang, Da Liu, Cong Peng, Dongshan Zhang, Xudong Xiang

**Affiliations:** ^1^Department of Respiratory Medicine, Hunan Centre for Evidence-Based Medicine, Research Unit of Respiratory Diseases, The Second Xiangya Hospital, Central South University, 139 Middle Renmin Road, Changsha, Hunan 410011, China; ^2^Department of Emergency, Institute of Emergency Medicine and Difficult Diseases, The Second Xiangya Hospital, Central South University, 139 Middle Renmin Road, Changsha, Hunan 410011, China; ^3^Department of Anesthesiology, Xiangya Hospital, Central South University, 87 Xiangya Road, Changsha, Hunan 410008, China; ^4^Department of Respiratory Medicine, The First Hospital of Guangyuan City, 490 Juguo Road, Guangyuan, Sichuan 628000, China; ^5^Department of Respiratory Medicine, Peace Hospital, Changzhi Medical College, Changzhi, Shanxi 046000, China; ^6^Department of the Second Thoracic Medicine, The Affiliated Cancer Hospital of Xiangya School of Medicine, Central South University, 283 Tongzipo Road, Changsha, Hunan 410006, China; ^7^Department of Respiratory Medicine, Hunan Provincial People's Hospital, 61 West Jiefang Road, Changsha, Hunan 410005, China; ^8^Department of Respiratory Medicine, The Second Hospital, University of South China, 30 Jiefang Road, Hengyang, Hunan 421001, China; ^9^Department of Respiratory Medicine, Changsha Central Hospital, 161 South Shaoshan Road, Changsha, Hunan 410004, China; ^10^Department of Dermatology and Venereology, Xiangya Hospital, Central South University, 87 Xiangya Road, Changsha, Hunan 410008, China

## Abstract

Th17 cells and IL-17 participate in airway neutrophil infiltration characteristics in the pathogenesis of severe asthma. Methyl-CpG binding domain protein 2 (MBD2) expression increased in CD4^+^ T cells in peripheral blood samples of asthma patients. However, little is known about that epigenetic regulation of MBD2 in both immunological pathogenesis of experimental severe asthma and CD4^+^ T cell differentiation. Here, we established a neutrophil-predominant severe asthma model, which was characterized by airway hyperresponsiveness (AHR), BALF neutrophil granulocyte (NEU) increase, higher NEU and IL-17 protein levels, and more Th17 cell differentiation. In the model, MBD2 and IRF4 protein expression increased in the lung and spleen cells. Under overexpression or silencing of the MBD2 and IRF4 gene, the differentiation of Th17 cells and IL-17 secretion showed positive changes. IRF4 protein expression showed a positive change with overexpression or silencing of the MBD2 gene, whereas there was no significant difference in the expression of MBD2 under overexpression or silencing of the IRF4 gene. These data provide novel insights into epigenetic regulation of severe asthma.

## 1. Introduction

Asthma is a complex chronic inflammatory disease characterized by AHR, reversible airflow obstruction, and airway inflammation [1]. IL-17 (also known as IL-17A) is a representative cytokine produced by Th17 cells, which can be induced by the release of IL-8, CXC, and other neutrophil chemokines to raise and activate neutrophils [2–4], and animal and clinical analysis of samples have proved that Th17 cells and IL-17 play important roles in the pathogenesis of asthma. These asthma cases are characterized by predominantly neutrophilic and mixed granulocytic types and are associated with severe asthma and poor response to corticosteroids [5–9]. It is therefore very important to establish a NEU predominant inflammatory phenotype asthma model, and we tried using 100 *μ*g of HDM and OVA combined with 15 *μ*g LPS to establish a neutrophil-predominant severe asthma model and to further study the relationship between the changes of Th17 cells and the epigenetic alterations.

MBD2 (methyl-CpG binding domain protein 2) can specifically bind to the promoter region of a target gene and change in the posttranscriptional modification of histone through the recruitment of other molecules, thus changing the chromatin structure and regulating the expression of target genes. In our preliminary study, we found that the expression of MBD2 increased obviously after accepting stimulus differentiation in splenic CD4^+^ T cell in mice. Through the analysis of peripheral blood samples of asthma patients, we also found that MBD2 expression increased in CD4^+^ T cells in peripheral blood compared with healthy people, meaning that MBD2 has a close relationship with both the immunological pathogenesis of asthma and CD4^+^ T cell differentiation. Therefore, MBD2 could play an important role in neutrophil-predominant severe asthma, and to find which cytokine it impacts is our next research goal.

IRF4 (interferon regulatory factor 4) is strictly expressed in immune cells. In IRF4 research on the role of Th17 cell differentiation, Brüstle found that mice knockout IRF4 failed to develop experimental autoimmune encephalomyelitis, T cell and RORgamma T were expressed lower, and Th17 cell differentiation was impaired [10]. So, IRF4 is therefore an important factor in neutrophil-predominant severe asthma.

At present, there is little research on the role of MBD2 in severe asthma, and the relationship between MBD2 and IRF4 in severe asthma is not known. Here, through the above data review, we hypothesize that MBD2 would regulate Th17 cell differentiation and experimental severe asthma by affecting IRF4.

## 2. Materials and Methods

### 2.1. Ethics Statement

All studies were performed in compliance with the Second Xiangya Hospital and Central South University Animal Care and Use Committee guidelines.

### 2.2. Mice

Female C57BL/6 mice (provided by animal center of the Second Xiangya hospital of Central South University, China), aged 6-7 weeks, weighing 18–20 grams, were used in the experiments. All mice were bred and housed in a SPF facility with a 12/12 h light/dark cycle.

### 2.3. Experimental Reagents and Equipment

The HDM were supplied by GREER; the LPS, aluminum hydroxide gel, and OVA were supplied by Sigma; small animal cough- and asthma-inducing instrument (YLS-8A) was purchased from Beijing Zhongshidichuang Science and Technology Development Co. Ltd.; mice spirometer (MAX 1320) was purchased from Buxco®, USA; low-speed centrifuge (5810 R) was purchased from Eppendorf®, Germany; optical microscope (DMI3000B) was from Olympus, Japan (Leica®); and pathological image analyzer (Leica Application Suite V4) was purchased from Leica, Germany.

### 2.4. Mice Asthma Model

Female C57BL/6 mice were grouped according to a random number table, with six mice in each group. The group of severe asthma model mice were given an intraperitoneal sensitization injection with 100 *μ*g HDM + 100 *μ*g OVA+ 15 *μ*g LPS + 2 mg aluminum hydroxide on days 0, 1, and 2 [11] and then challenged with OVA solution atomized for 30 minutes before HDM intranasal excitation on days 14, 15, 18, and 19. For the saline group, mice received saline only. The group of conventional asthma model mice were given a sensitization intraperitoneal injection with 25 *μ*g OVA + 1 mg aluminum hydroxide on days 0 and 7 and then challenged with OVA solution atomized excitation for 30 minutes on days 14, 15, 16, 17, 18, 19, and 20 [12]. Mice were sacrificed on day 21.

### 2.5. Ethology Observed

Mice ethology was observed everyday as follows: fur luster, nose and ear scratching, irritability, sneezing, rapid breathing, and incontinence.

### 2.6. Airway AHR

Methacholine- (Mch-) induced airway resistance was measured on day 21 (24 h after the final challenge) by direct plethysmography (Buxco Electronics, RC System, USA). The procedures were the same as previously described [13]. Mice were anesthetized, tracheotomized, and then intubed. At first, the baseline of lung resistance (RL0) was recorded for one minute. Then, mice were given 10 *μ*l saline and 10 *μ*l Mch with increasing doses of 0.39 mg/ml (dose 1), 0.78 mg/ml (dose 2), 1.56 mg/ml (dose 3), and 3.12 mg/ml (dose 4) atomized to stimulate the airway, and the changes of lung resistance (RLX) were then recorded. The ratios of RLX/RL0 were used as the final results for analysis.

### 2.7. BALF Cell Count

BALF collection procedures were the same as previously described [13]. After eliminating red blood cells, by centrifugation and precipitation, total BALF cell counts were determined by a haemocytometer. Then, cell slices were made using a Biping settlement system. BALF NEU and EOS cell counts were determined in 200 total BALF cells after cell slices underwent H&E staining.

### 2.8. Histopathological Analysis of Inflammatory and Structural Changes

The right lungs were incubated in 4% paraformaldehyde for 24 h and level dehydration: 70% ethanol (5 min), 75% ethanol (5 min), 80% ethanol (5 min), 90% ethanol (5 min), 95% ethanol (5 min), 100% ethanol Ι (10 min), 100% ethanol II (10 min), xylene liquid Ι (10 min), and xylene liquid II (10 min) and then embedded in paraffin.

#### 2.8.1. Lung Tissue Inflammation Score

Lung tissues were stained with H&E, and 3~4 H&E stained sections were chosen per group under a single-blind procedure. Two pathologists assessed the lung tissue inflammation score under a microscope as previously described [14]: a score of 0 represented no inflammatory cell infiltration; a score of 1 represented little inflammatory cell infiltration; a score of 2 represented 1 layer of inflammatory cells around the airway; a score of 3 represented 2~4 layers of inflammatory cells around the airway; and a score of 4 represented 4 or more layers of inflammatory cells around the airway.

#### 2.8.2. Immunohistochemistry for NEU, EOS, IL-17A, IL-4, MBD2, and IRF4

Lung tissues were stained for immunohistochemistry (neutrophil-specific antibody (anti-Gr1, Biolegend), eosinophil antibody (anti-ECP, Biorbyt), IL-17A antibody (Proteintech), IL-4 antibody (ABBIOTEC), MBD2 antibody (Abcam), and IRF4 antibody (Proteintech)), and 2~4 H&E stained sections per group were chosen under single-blind conditions to detect and localize NEU, EOS, IL-17A, IL-4, MBD2, and IRF4 protein expression. Using a microscope and computer image processing system (Image-Pro Plus 6.0), two pathologists assessed the NEU, EOS, IL-17A, IL-4, MBD2, and IRF4 scores.

### 2.9. Western Blotting

Total proteins were prepared using RIPA lysis buffer supplemented with protease inhibitors. Western blotting was carried out by probing the membranes with indicated primary antibodies followed by incubating with an HRP-conjugated secondary antibody. The NEU, EOS, IL-17A, IL-4, MBD2, and IRF4 antibodies were the same as above.

### 2.10. Bronchial Lung Tissue Suspension Cells

Mice bronchial lungs were collected, washed once with 5x antibiotic, and washed 2 times with PBS. Then, the lungs were cut as little as possible, and subsequently, digestive juice consisting of collagenase1 (0.5 mg/ml Sigma, unit/ml) + 10 *μ*g/ml DNase in RPMI medium was added and kept for 1 hour at 37°C in a water bath shaking incubator. After the digestion of the lung tissue and straining with a 70 *μ*m filter Cell Strainer (BD Falcon), cells were centrifugated and resuspended in 10% FBS culture medium.

### 2.11. T Cell Purification, Activation, and Staining

Spleen CD4^+^ T cells of model mice were selected by using microbead sorting (130-049-201, Miltenyi Biotec, Germany) and were then seeded in 12-well flat bottom plates. In the next 5 hours, the cells were restimulated with 50 ng/ml phorbol-12-myristate-13-acetate (PMA) (Multi Sciences Company, China), 1 *μ*g/ml ionomycin (Multi Sciences Company, China), and 3 *μ*g/ml monensin (Multi Sciences Company, China). The lung cells were then stained for surface marker FITC-antiCD4 cytokine antibody (BioLend, USA) followed by fixation and permeabilization with fixation and permeabilization buffer (Multi Sciences Company, China) 15 min. After washing with permeabilization buffer, the lung and spleen cells were stained with intracellular markers APC-anti-IL-17 and PE-anti-IL-4 cytokine antibodies (BioLend, USA) in the permeabilization buffer for 20 min. Flow cytometry was conducted and the data were analyzed using the FACSCalibur and FlowJo version X software.

### 2.12. Th17 Cell Differentiation

For directed differentiation of Th17 cells, 1 × 10^6^ CD4^+^ T cells from C57BL/6 mice were activated with 2 *μ*g/ml anti-CD3, 2 *μ*g/ml anti-CD28 for 3 days in the presence of 5 ng/ml TGF-beta, 20 ng/ml IL-6 and 20 ng/ml IL-23. For neutralization of IL-4 and IFN-gamma, 10 *μ*g/ml anti-IL-4 and 10 *μ*g/ml anti-INF-gamma were added into the cultures [15, 16]. All cytokines were purchased from BioLend Co. (BioLend, USA).

### 2.13. Lentiviral Transduction of Th17 Cells

Splenic CD4^+^ Th17 lymphocytes were transfected with chemosynthesis MBD2 siRNA sequence (S) 5′-GTTTGGCTTAACACATCTCAA-3′ and IRF4 siRNA sequence (S) 5′-GCCAGACAACTGTATTACTTT-3′. The Th17 cells were cultured in 1640 medium without serum. The cells were resuspended according to 1.5 × 10^6^/tube and MOI = 20, and the appropriate amount of virus was used for transfection. The cells were seeded in 12-well flat-bottom plates for 72 h. In the last 5 h, the cells were restimulated with PMA, ionomycin, and monensin as above.

## 3. Results

### 3.1. The Severe Asthma Mice Model Was Established

**Figure 1:**
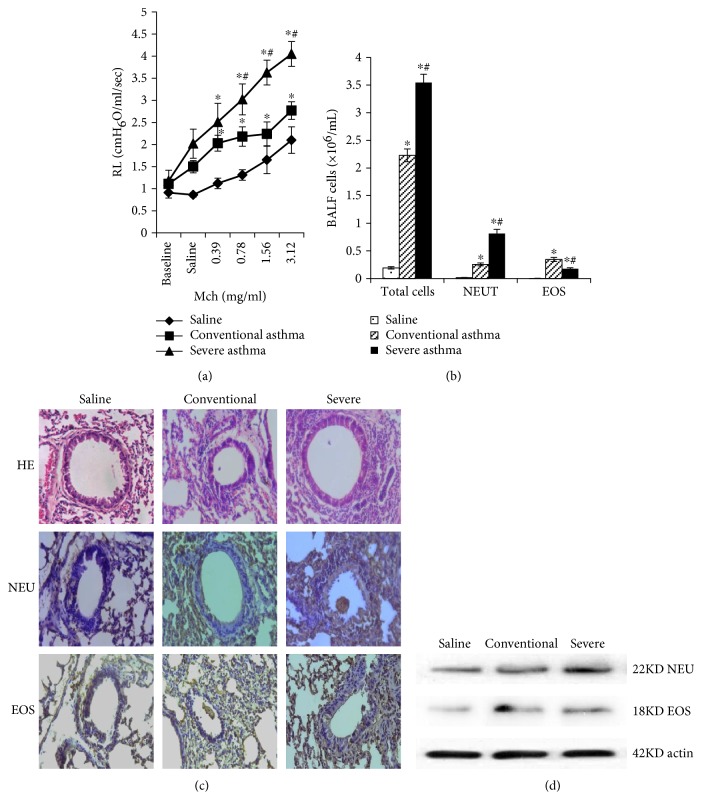
The severe asthma mice model was established. (a) Pulmonary resistance in the saline, conventional, and severe groups. When Mch was inhaled, the RL of the conventional and severe groups showed a rise, and the RL values of the severe group were higher than those of the conventional group. (b) The total, NEU, and EOS cells of BALF from the three groups. The total and NEU cells of BALF from the severe group were higher than those from the conventional group, but EOS cells showed an opposite result. (c) Lung tissues were stained with H&E, immunohistochemistry (neutrophil-specific antibody (anti-Gr1), eosinophil antibody (anti-ECP)) of the three groups. Histological analyses of lungs from the severe group exhibited markedly enhanced peribronchial inflammation with infiltrated neutrophils, but eosinophils were not obviously different compared with the conventional group. (d) Western blot analysis detected NEU and EOS protein expression in the three groups. The NEU protein expression of the lungs from the severe group were higher than those from the conventional group, whereas EOS protein expression values were not obviously different between them. ^∗^*p* < 0.05 as compared with the control group. ^#^*p* < 0.05 as compared with the conventional group.

We first observed sleepiness in severe asthma mice on the 4th day of stimulation, compared to the 5th day for conventional asthma mice. When Mch was inhaled, the pulmonary resistance (RL) of the severe group showed a sharp rise (Figure 1(a)). Through BALF of the three groups, we found that total cell counts and neutrophil granulocyte counts of the severe group were the highest, but the eosinophil granulocyte counts of the conventional group were the most (Figure 1(b)). Histological analyses of lungs from the severe group exhibited markedly enhanced peribronchial inflammation with infiltrated neutrophils, but eosinophils were not obviously different compared with the conventional group (Figure 1(c)). Results of Western blot analyses were the same as those of histological analyses (Figure 1(d)).

### 3.2. The Severe Asthma Mice Was Mediated by Th17 Cells

**Figure 2:**
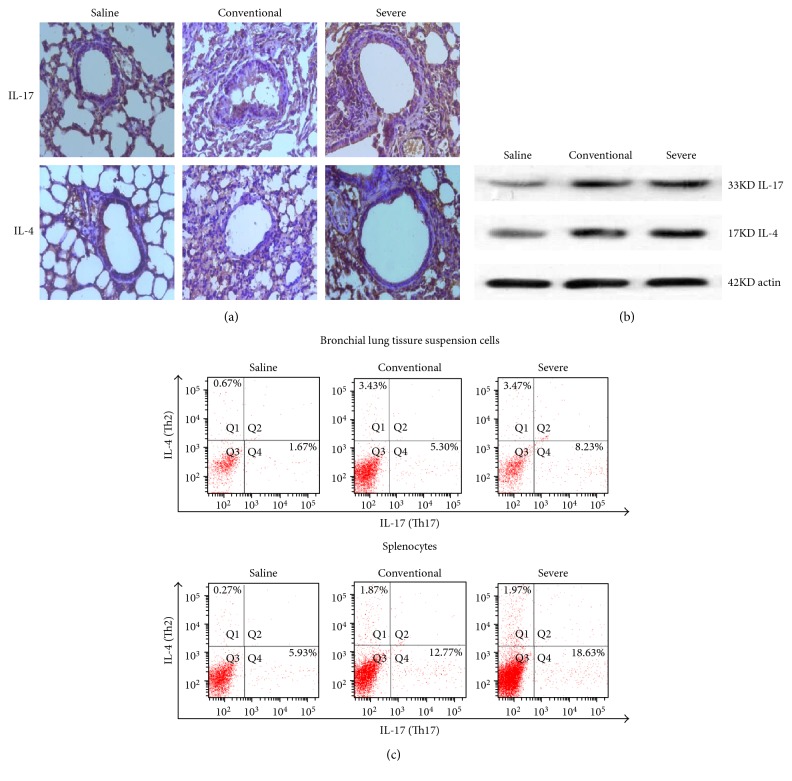
The severe asthma mice were mediated by Th17 cells. (a) Lung tissues were stained for immunohistochemistry (anti-IL-17, anti-IL-4) of the three groups. Histological analyses of lungs from the severe group exhibited markedly enhanced cells with stained IL-17 positively, but cells with stained IL-4 positively were not obviously different compared with those from the conventional group. (b) Western blot analysis detected IL-17 and IL-4 protein expression in the three groups. The IL-17 protein expression of the lungs from the severe group was higher than that from the conventional group, whereas IL-4 protein expression values were not obviously different between them. (c) Th17 and Th2 cells were tested in bronchial lung tissue suspension cells and splenocytes, and the cells were then subjected to intracellular staining of APC-anti-IL-17 and PE-anti-IL-4 by flow cytometry analyses. Th17 cells were the most in bronchial lung tissue suspension cells and splenocytes from the severe group compared with the conventional group, whereas Th2 cells were not obviously different between them.

IL-17 is the main representative cell factor of Th17 cells, as is IL-4 for Th2 cells. Histological analyses of lungs from the severe group exhibited markedly enhanced cells with stained IL-17, but cells with stained IL-4 were not obviously different compared with the conventional group (Figure 2(a)). Results of Western blot analyses were the same as histological analyses (Figure 2(b)). Th17 cells were the most in bronchial lung tissue suspension cells and splenocytes from the severe group compared with the conventional group (Figure 2(c)).

### 3.3. Expression of MBD2 in Severe Asthma Mice

**Figure 3:**
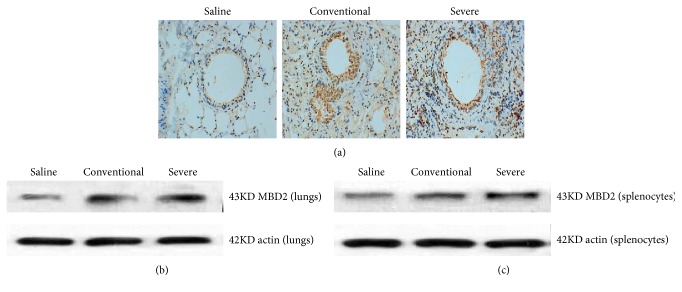
Expression of MBD2 in three groups. (a) Lung tissues were stained for immunohistochemistry (anti-MBD2) of the three groups. Histological analyses of lungs from the severe group exhibited more cells with stained MBD2 positively than those from the conventional group. (b) Western blot analyses detected MBD2 protein expression in the lungs of the three groups. The MBD2 protein expression of the lungs from the severe group was higher than that from the conventional group. (c) Western blot analyses detected MBD2 protein expression in the splenocytes of the three groups. The MBD2 protein expression of the lungs from the severe group was higher than that from the conventional group.

Histological analyses of lungs from the severe group exhibited markedly enhanced cells with stained MBD2 compared with the conventional group (Figure 3(a)). MBD2 expression in lungs and splenic CD4^+^T cells from the severe group were significantly increased compared with the conventional group (Figures 3(b) and 3(c)).

### 3.4. IL-17 Expression and Th17 Cell Differentiation under MBD2 Gene Silencing or Overexpression

**Figure 4:**
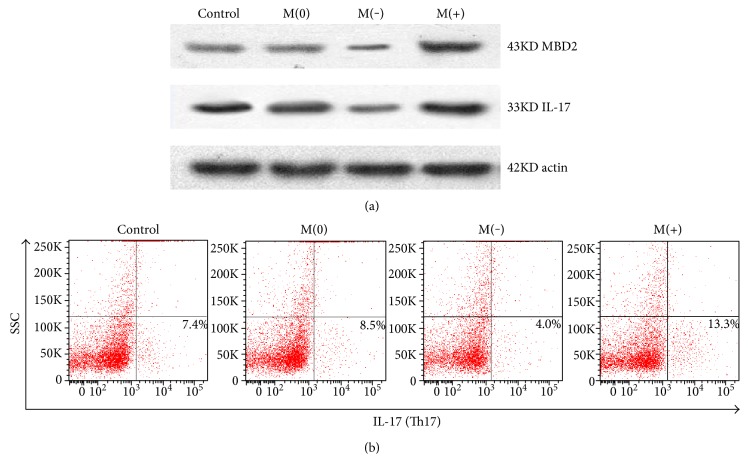
IL-17 expression and Th17 cell differentiation under MBD2 gene silencing or overexpression. (a) Under MBD2 gene silencing (M(−)), IL-17 and MBD2 protein expression was significantly lower than that of the empty transfection group (M(0)) by Western blot analyses; with MBD2 gene overexpression (M(+)), IL-17 and MBD2 protein expression was markedly increased than that of M(0). (b) Under MBD2 gene silencing (M(−)), Th17 cell differentiation was significantly lower than that of M(0) according to flow cytometry analyses; with MBD2 gene overexpression (M(+)), Th17 cell differentiation was markedly increased compared to that of M(0).

We conducted Western blot analyses and demonstrated either MBD2 gene silencing (M(−)) or overexpression (M(+)) in splenic CD4^+^T cells successfully. With MBD2 gene silencing, IL-17 expression was significantly lower than that of the empty transfection group (M(0)); and when the MBD2 gene was overexpressed, IL-17 expression was markedly increased compared to that of the empty transfection group (Figure 4(a)). Th17 cells were obviously decreased or increased under MBD2 gene silencing or overexpression (Figure 4(b)).

### 3.5. Expression of IRF4 in Severe Asthma Mice

**Figure 5:**
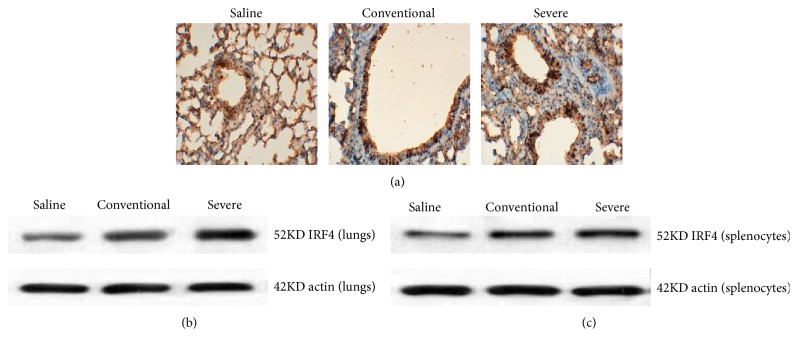
Expression of IRF4 in three groups. (a) Lung tissues were stained for immunohistochemistry (anti-IRF4) of the three groups. Histological analyses of lungs from the severe and conventional groups exhibited markedly enhanced cells with stained IRF4 compared with those of the saline group. (b) Western blot analyses detected IRF4 protein expression in the lungs of the three groups. The IRF4 protein expression from the severe and conventional groups were higher than that from the saline group. (c) Western blot analyses detected IRF4 protein expression in the splenocytes of the three groups. The IRF4 protein expression from the severe and conventional groups were higher than that from the saline group.

Histological analyses of the lungs from the severe and conventional groups exhibited markedly enhanced cells with stained IRF4 compared with those of the saline group (Figure 5(a)). IRF4 expression in the lungs and splenic CD4^+^T cells from the two groups were significantly increased compared with that of the saline group, but there was no significant difference between the two groups (Figures 5(b) and 5(c)).

### 3.6. IL-17 and MBD2 Expression under IRF4 Gene Silencing or Overexpression

**Figure 6:**
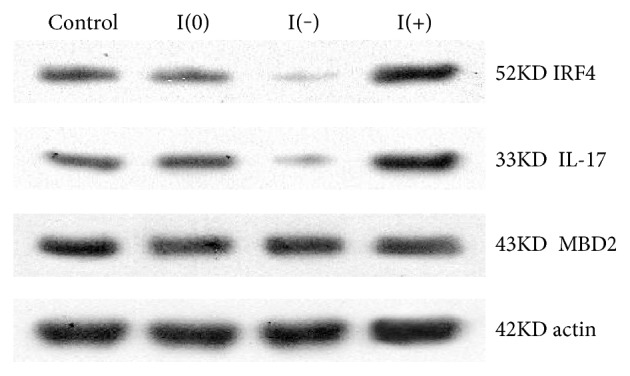
IL-17 and MBD2 expression under IRF4 gene silencing (I(−)) or overexpression (I(+)). Under I(−), IL-17 and IRF4 protein expression were significantly lower than that of the empty transfection group (I(0)) according to Western blot analyses; under I(+), IL-17 and IRF4 protein expression were markedly increased compared to that of I(0). Under I(−) or I(+), MBD2 protein expression showed no significant difference.

We demonstrated that IRF4 gene silencing (I(−)) or overexpression (I(+)) in splenic CD4^+^T cells was successful. Under IRF4 gene silencing, IL-17 expression was significantly lower than that of the empty transfection group (I(0)), and under IRF4 gene overexpression (I(+)), IL-17 expression was markedly increased compared to that of the empty transfection group. Under IRF4 gene silencing or overexpression, no significant difference in MBD2 expression was observed (Figure 6).

### 3.7. IL-17 Expression and Th17 Cell Differentiation under Joint MBD2 and IRF4 Gene Silencing or Overexpression

**Figure 7:**
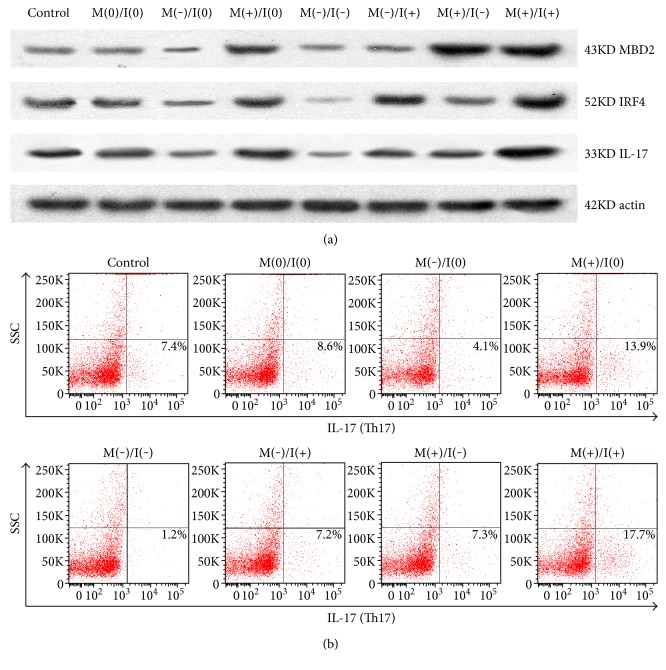
IL-17 expression and Th17 cell differentiation under joint MBD2 and IRF4 gene silencing or overexpression. (a) Under joint M(−)/I(−), IL-17 protein expression was significantly the lowest according to Western blot analyses; under joint M(−)/I(0), IL-17 protein expression was higher than M(−)/I(−). While under joint M(+)/I(+), IL-17 protein expression was significantly the highest; under joint M(+)/I(0), IL-17 protein expression was lower than M(+)/I(+). IL-17 protein expression showed no significant difference between the control group, M(0)/I(0), M(−)/I(+), and M(+)/I(−). (b) Under joint M(−)/I(−), Th17 cell differentiation was significantly the lowest according to flow cytometry analyses; under joint M(−)/I(0), Th17 cell differentiation was higher than M(−)/I(−). Under joint M(+)/I(+), Th17 cell differentiation was significantly the highest; under jointM(+)/I(0), Th17 cell differentiation was lower than M(+)/I(+). No significant difference in Th17 cell differentiation was observed between the control group, M(0)/I(0), M(−)/I(+), and M(+)/I(−).

We found that IL-17 expression was significantly the lowest when both MBD2 and IRF4 underwent joint gene silencing, while IL-17 expression was the highest when both MBD2 and IRF4 underwent joint gene overexpression (Figure 7(a)). Th17 cells were obviously the least or the most while both MBD2 and IRF4 underwent joint gene silencing or overexpression (Figure 7(b)).

## 4. Discussion

In order to better study the mechanisms of severe asthma, we urgently need to build a neutrophil-predominant severe asthma model. While OVA is a classic asthma model allergen, exposure to LPS is also known to lead to an increased risk of asthma-like symptoms [17] and the onset of asthma exacerbations [18, 19]. Meanwhile, the development of allergic asthma is strongly associated with the exposure to HDM [20, 21]. When OVA-induced asthmatic mice are re-exposed to HDM, the pathomechanism is different from OVA exposure alone [22]. Although there have been lots of similar experiments, there are so little successful severe asthma models. In our previous study, we tried different doses of HDM and OVA combined with 15 *μ*g LPS to establish a neutrophil-predominant severe asthma model and found that 100 *μ*g HDM + 100 *μ*g OVA + 15 *μ*g LPS successfully established a NEU predominant inflammatory phenotype severe asthma model. Here, compared with the conventional asthma mice sensitized by OVA only (named the OVA group), among BALF total cell counts, BALF NEU was significantly increased in the severe asthma mice sensitized by HDM/OVA/LPS, but BALF EOS showed an opposite tendency. Similarly, analysis of lung tissue sections revealed that the HDM/OVA/LPS sensitized group caused a significant increase of both histological inflammation scoring and NEU expression compared to the OVA group, whereas there was no difference in the expression of EOS between the two groups. Compared to moderate asthma, IL-4 expression was decreased [7] and IL-17A expression was increased [23, 24] in severe asthma. In the present study, lung tissue IL-17A expression was significantly increased in the HDM/OVA/LPS sensitized group when compared with the OVA group, whereas there was no difference in the expression of IL-4 between the two groups. The spleen is the classical observation target for immune cells, and Th17 cells were the most in the splenocytes from severe asthma mice compared with conventional asthma mice according to flow cytometry. The lung is one of the effector organs of immunity, and Th17 cells were the most in the lung tissue suspension cells from severe asthma mice compared with conventional asthma mice according to flow cytometry. This showed that neutrophilic predominant severe asthma mice first exhibited mainly Th17 cell differentiation followed by an increase of IL-17 secretion, and the recruitment of NEU, thus promoting the development of asthma, in a way distinctly different from the classic EOS allergic asthma involving IL-4 recruitment. Therefore, the neutrophil-predominant severe asthma model was established successfully.

MBD2, as an epigenetic regulation element [25], can regulate T cells to differentiate into Th17 cells by methylating T-bet/Hlx [26], so it could be involved in the pathogenesis of severe asthma. Considering that Th17 cell is mainly involved in the inflammatory response, after being induced by oligodendrocytes, EAE mice would develop severe paralysis, but MBD2 null mutant mice were completely protected from EAE induction [26]. In our study, MBD2 showed significantly increased expression in the splenocytes of the severe asthma model mice, representing the classical observation target as immune cells. The lung is one of the effector organs of immunity, and MBD2 showed a higher expression in the lung tissue of severe asthma model mice compared with the conventional asthma model mice. To ascertain whether MBD2 is involved in the pathogenesis of severe asthma, we conducted in vitro splenocyte experiments and found that IL-17 protein expression increased significantly along with overexpression of the MBD2 gene and decreased with the silencing of the MBD2 gene. At the same time, the number of Th17 cells showed a consistent change with the overexpression or silencing of the MBD2 gene. Through these tests, MBD2 is shown to participate in severe asthma by affecting the differentiation of Th17 cells and IL-17 secretion.

Next, we needed to analyze whether MBD2 can affect IRF4 and participate in asthma. IRF4, as an essential controller of Th17 differentiation, participates in many immune diseases [27]. IRF4-deficient mice are totally resistant to the development of a Th17-mediated disease [10]. Overexpressed IRF4 in naive CD4^+^ T cells derived from relapsing remitting multiple sclerosis patients significantly increased their ability to secrete IL-17A, IL-17F, IL-21, and IL-22 [28]. We found that both IRF4 and IL-17 had a significantly increased expression in the lung and the spleen cells of severe asthma model mice. From the in vitro cell experiment, IL-17 protein expression increased significantly with overexpression of the IRF4 gene and decreased with the silencing of the IRF4 gene. These findings show that IRF4 participates in severe asthma by affecting the differentiation of IL-17 secretion and is consistent with previous studies. Then, from the in vitro splenocyte experiment, we found that IRF4 protein expression increased significantly along with overexpression of the MBD2 gene and decreased with the silencing of the MBD2 gene, whereas there was no significant difference in the expression of MBD2 under overexpression or silencing of the IRF4 gene. This shows that MBD2 can affect IRF4 expression in severe asthma.

Finally, in conditions of joint overexpression of both MBD2 and IRF4 genes, the levels of expression of IRF4, differentiation of Th17 cells, and IL-17 secretion were the highest, while joint silencing of MBD2 and overexpression of IRF4 genes, the levels of expression of IRF4, differentiation of Th17 cells, and IL-17 secretion were declined. Under conditions of joint silencing of both genes, the levels of expression of IRF4 and differentiation of Th17 cells and IL-17 secretion were the lowest, while joint overexpression of MBD2 and silencing of IRF4 genes, the levels of expression of IRF4, and differentiation of Th17 cells and IL-17 secretion were increased. This indicates that MBD2 can participate in the differentiation of Th17 cells and IL-17 secretion through IRF4 expression and so participate in the pathogenesis of the neutrophil-predominant severe asthma model.

## Abbreviations

MBD2: Methyl-CpG binding domain protein 2
BALF: Bronchoalveolar lavage fluid
AHR: Airway hyperresponsiveness
IRF4: Interferon regulatory factor 4
HDM: House dust mite
OVA: Ovalbumin
LPS: Lipopolysaccharide
NEU: Neutrophil granulocyte
EOS: Eosinophilic granulocyte
RL: Pulmonary resistance.
